# Specific inhibition of acetylcholinesterase as an approach to decrease muscarinic side effects during *myasthenia gravis* treatment

**DOI:** 10.1038/s41598-017-18307-9

**Published:** 2018-01-10

**Authors:** Konstantin A. Petrov, Alexandra D. Kharlamova, Oksana A. Lenina, Ayrat R. Nurtdinov, Marina E. Sitdykova, Victor I. Ilyin, Irina V. Zueva, Evgeny E. Nikolsky

**Affiliations:** 1 A.E. Arbuzov Institute of Organic and Physical Chemistry of Russian Academy of Sciences, Arbuzov str. 8, Kazan, 420088 Russia; 2 Kazan Federal University, 18 Kremlyovskaya str, Kazan, 420008 Russia; 3 Kazan State Medical University, 49 Butlerova Street, Kazan, 420012 Russia; 4 Kazan Institute of Biochemistry and Biophysics of Russian Academy of Sciences, Lobachevsky str. 2/31, Kazan, 420111 Russia

## Abstract

Non-selective inhibitors of cholinesterases (ChEs) are clinically used for treatment of *myasthenia gravis* (*MG*). While being generally safe, they cause numerous adverse effects including induction of hyperactivity of urinary bladder and intestines affecting quality of patients life. In this study we have compared two ChEs inhibitors, a newly synthesized compound C547 and clinically used pyridostigmine bromide, by their efficiency to reduce muscle weakness symptoms and ability to activate contractions of urinary bladder in a rat model of autoimmune *MG*. We found that at dose effectively reducing *MG* symptoms, C547 did not affect activity of rat urinary bladder. In contrast, at equipotent dose, pyridostigmine caused a significant increase in tonus and force of spontaneous contractions of bladder wall. We also found that this profile of ChEs inhibitors translates into the preparation of human urinary bladder. The difference in action observed for C547 and pyridostigmine we attribute to a high level of pharmacological selectivity of C547 in inhibiting acetylcholinesterase as compared to butyrylcholinesterase. These results raise reasonable hope that selective acetylcholinesterase inhibitors should show efficacy in treating *MG* in human patients with a significant reduction in adverse effects related to hyperactivation of smooth muscles.

## Introduction

Synaptic transmission at the skeletal neuromuscular junction (NMJ) is indispensable for survival of living organisms by transducing complexity of cerebral commands to muscular twitches. In the vertebrate NMJ presynaptic electrical signal is transmitted by acetylcholine (ACh) which is released from motor nerve ending, and then diffuses through synaptic cleft to activate postsynaptic nicotinic acetylcholine receptors (nAChRs) of muscle type ((α1)2β1δε)^1^. This, in turn, leads to membrane depolarization (postsynaptic excitatory potential), triggering action potential (AP) and muscle twitch. Impairment of neuromuscular synaptic transmission results in muscle weakness and even death if synapses of respiratory muscles are affected.

The most common form of pathological muscle weakness is *myasthenia gravis* (*MG*). This chronic autoimmune neuromuscular disorder is characterized by fluctuating weakness of voluntary muscle groups. Weakness in *MG* is caused by autoantibodies directed specifically and primarily towards muscle type nAChRs. Antibodies reduce the number of functional nAChRs in the NMJs by a combination of complement-mediated membrane lysis and acceleration of receptor catabolism^2,3^. The cause of autoimmune response is unknown and only symptomatic therapies for *MG* are currently available. Clinically relevant treatments of *MG* include immunosuppressive drugs, plasmapheresis, thymectomy and inhibitors of cholinesterases (ChEs)^4^. All treatments suffer from a variety of side effects. For daily pharmacological correction of muscle weakness, the most frequently used drugs cause partial inhibition of AChE and butyrylcholinesterase (BChE). These enzymes catalyze hydrolysis of ACh, thus terminating its action on ACh receptors^5^. Extension of duration of ACh action at partial inhibition of ChEs is able to compensate for autoimmune decrease in nAChRs density and, thus, rescues muscle contractions. Inhibition of ChEs at the NMJs seems to be sufficient for therapeutic efficiency of esterase inhibitors used in *MG* treatment (but see Discussion for consequences of inhibition of BChE in NMJs of skeletal muscles). However, inhibition of ChEs in other tissues also occurs resulting in adverse effects. Significant bulk of side effects is associated with hyperactivation of muscarinic acetylcholine receptors (mAChRs) in vegetative nerve system, primarily in smooth muscles and, to a lesser extent, in myocardium^6,7^.

Previously we have described a series of cholinesterase inhibitors based on 1,3-bis[5-(*o*-nitrobenzylethylammonium)pentyl]-6-methyluracilic unit with selectivity towards mammalian AChE *vs*. BChE^8–11^. These inhibitors were found to be efficacious in an animal model of *MG* and can be considered as potentially valuable candidates for treatment of pathological muscle weakness syndromes in humans. Recently, the most selective compound, 6-methyluracil derivative, C547, was pharmacologically profiled on human AChE and BChE. Kinetic analysis of inhibition showed that C547 is a slow-binding inhibitor of type B, i.e. after formation of initial enzyme-inhibitor complex (*K*_i_ = 140 pM), it slowly transits into the final equilibrium high-affinity state (*K*_i_* = 22 pM). On the other hand, on human BChE, C547 is a fast-binding reversible inhibitor of mixed-type (*K*_i_ = 1.77 μM; *K*_i_’ = 3.17 μM)^12,13^. Thus, C547 was found to be one of the most potent and selective reversible inhibitors of AChE discovered so far.

In this study, we decided to compare effects of C547 and clinically used non-selective ChEs inhibitor, pyridostigmine, on contractility of rat and human urinary bladder muscle preparations. We found that C547, in the doses effectively controling muscle weakness in *in vivo* experiments, did not affect activity of rat bladder muscles. In contrast, the dose of pyridostigmine required to alleviate *MG* symptoms enhanced the tonus of urinary bladder and significantly amplified the force of its spontaneous contractions. We assume, that the difference in the effectivness of inhibitors is due to higher selectivity of C547 with respect to AChE as compared to BChE. Our experiments allow us to suggest that, after partial and selective inhibition of AChE, remaining activity of BChE in the urinary bladder is sufficient to prevent development of significant side effects. We also made an important finding that sensitivity of human urinary bladder preparations to AChE and BChE inhibition is similar to that of the rat bladder. This observation provides reasonable bases to hypothesize that remaining activity of BChE in urinary bladder in humans can also be sufficient to reduce side effects when selective AChE inhibitors are used for *MG* treatment.

## Results

### Comparison of miniature end-plate currents in normal and myasthenic rats

Experimental autoimmune myasthenia gravis (EAMG) induced in rats, in its chronic phase, resembles human myasthenia. In our experiments, EAMG was induced by rat immunization with a peptide analogous to an amino acid sequence derived from α-subunit of rat muscle type nAChRs. As it has been shown earlier^14,15^, this type of rat EAMG resembles human myasthenia in the following aspects: (a) blood serum of affected animals contains antibodies toward muscle type nAChR; (b) there is a characteristic decrement in the amplitude of compound muscle AP (as evidenced by EMG) upon repetitive nerve stimulation as compared to normal animals. In addition to this, myasthenia should manifest itself by a decrease in amplitude and duration of miniature end-plate currents (mEPCs) as a result of the drop in the density of expressed and functional muscle nAChRs. These changes in mEPCs are most pronounced in “fast” muscles, e.g. extensor digitorum longus (EDL) muscle^16^. We decided to check-up whether these changes could be also observed in the EAMG model chosen. For this purpose we have compared mEPC recordings in normal animals and rats immunized with the peptide and developing characteristic decrement in the amplitude of compound muscle AP.

Mean values of resting membrane potential in EDL muscle fibers were essentially the same in healthy (−71 ± 2 mV, n = 30 fibers) and EAMG-affected animals (−70 ± 5 mV, n = 30 fibers).

**Figure 1:**
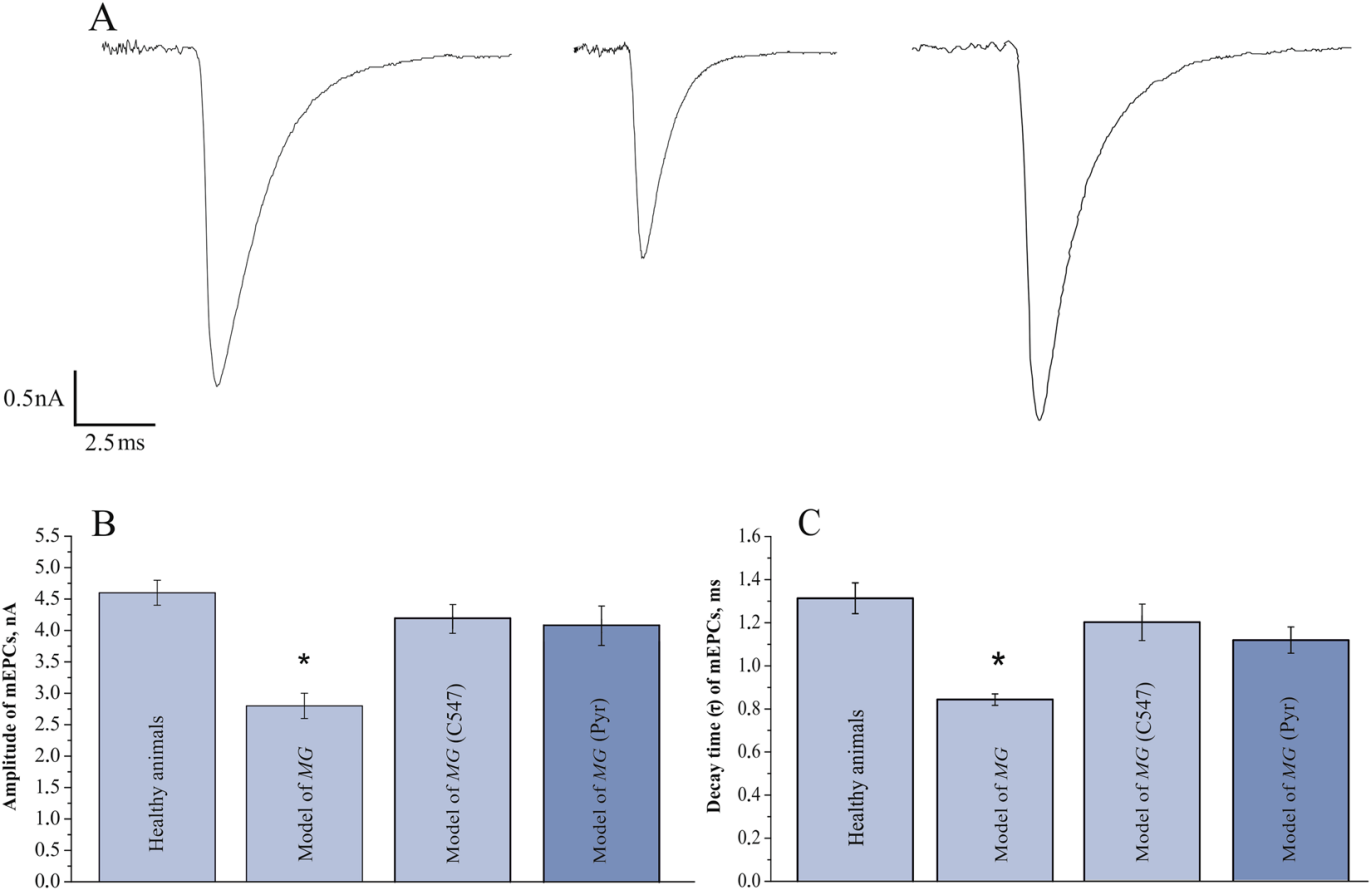
Amplitude and decay time constant of miniature end-plate currents are decreased at the NMJs in rats affected by EAMG. (**A**) Average of 100 individual miniature end-plate currents (mEPCs) recorded at the NMJ of healthy rat (left), rat affected by EAMG (middle) and EAMG-affected rat treated by 2 nM C547 (right). (**B**) Mean amplitude of mEPCs recorded in EDL muscles of healthy rats and animals affected by EAMG before and after inhibition of cholinesterases by C547 (2 nM) or pyridostigmine (Pyr, 1 µМ). (**C**) Mean decay time constant of mEPCs recorded in healthy rats and animals affected by EAMG before and after inhibition of cholinesterases by C547 (2 nM) or pyridostigmine (Pyr, 1 µМ). Data is presented as mean ± SEM pooled from five healthy rats and five EAMG-affected rats (5–6 end-plates *per* animal). Asterisk (*) indicates statistically significant difference (p < 0.05). The membrane potential was held at −60 mV.

We found that in normal muscle, the mean amplitude of mEPCs was equal to 4.6 ± 0.2 nA (n = 30 end-plates) and the mean time constant of current decay was 1.31 ± 0.72 ms (n = 30 end-plates). In diseased rats, both the amplitude and the duration of mEPCs were significantly reduced by 40% (2.8 ± 0.2 nA; n = 25 end-plates; p = 0.01, Student’s t-test) and 36% (0.84 ± 0.03 ms; n = 25 end-plates; p = 0.01, Student’s t-test), respectively (Fig. 1).

These results provide an electrophysiological proof of reduction of nAChR density in skeletal muscle NMJs in the EAMG model chosen.

### Effects of C547 and pyridostigmine on miniature end-plate currents in myasthenic rats

We were interested to see whether inhibition of ChEs can affect the parameters of mEPCs in EAMG-affected animals. We have compared effects of C547 and pyridostigmine at concentrations, which caused full block of AChE or AChE and BChE.

After 30 min pre-incubation with C547, 2 nM amplitude and duration of mEPCs restored practically to control values, i.e. to 4.2 ± 0.2 nA and 1.20 ± 0.08 ms, respectively (n = 25 end-plates, Fig. 1). Pre-incubation with pyridostigmine, 1 µМ caused similar effect, restoring mEPC amplitude and duration to 4.2 ± 0.3 nA and 1.10 ± 0.05 ms respectively (n = 25 end-plates, Fig. 1).

This observation is consistent with what one should expect from inhibition of synaptic ChEs: an increase in amplitude and duration of synaptic currents due to repetitive activation of nAChRs^17^.

### Effects of cholinesterase inhibition on symptoms of skeletal muscle weakness and on urinary bladder contractions in EAMG rat model

Previously we have tested C547 and pyridostigmine in the peptide-induced EAMG model in rat to find doses which reduce the decrement in the amplitude of compound muscle AP to control level. These doses for C547 and pyridostigmine were found to be 8 µg/kg, IP, and 100 µg/kg, IP, respectively^10^.

In this study, we decided to evaluate the effects of ChEs inhibitors on urinary bladder contractions in diseased animals. Spontaneous contractions of the urinary bladder in animals after EAMG development, diagnosed by electromyogram (EMG), were recorded. After that, rats were IP injected with C547 or pyridostigmine bromide and 20 minutes after, EMG and bladder contractions were recorded again.

**Figure 2:**
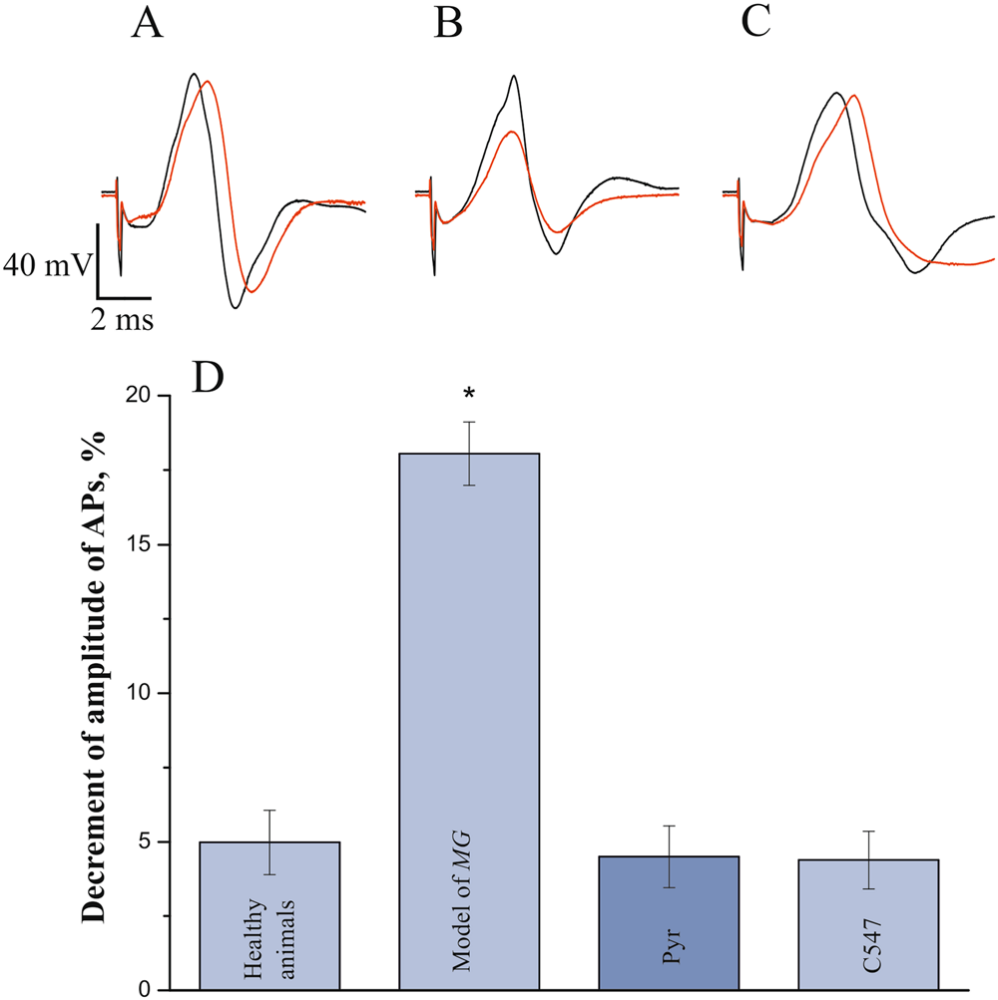
Restoration of decrement in amplitude of compound muscle action potential (AP) in EAMG rat upon specific inhibition of AChE and subsequent inhibition of BChE. (**A–C**) Electromyographic traces of the 1st (black line) and the 200th (red line) action potentials in normal muscles (**A**), in muscles affected by EAMG (**B**) and after C547 administration in EAMG rat (**C**). (**D**) Mean decrement of amplitude of APs recorded in healthy rats, in EAMG-affected rats and after IP administration of pyridostigmine (Pyr; 0.1 mg/kg) or C547 (0.008 mg/kg). Data are presented as mean ± SEM pooled from ten animals in each group. Asterisk (*) indicates statistically significant difference (p < 0.05).

EMG recordings from hindlimb muscles showed that EAMG resulted in a reduction in the amplitude of compound APs as measured by repetitive sciatic nerve stimulation (train of 200 electric pulses applied at 40 Hz). In healthy rats (n = 10 animals) amplitude of the 200th AP (AP200) in the train was 95 ± 1% of the 1st AP (AP1) (Fig. 2). In animals with EAMG (n = 20 rats, p = 0.01, Mann-Whitney test), AP200 was reduced to 80 ± 2% of control (Fig. 2). 20 min after IP administration, C547, 8 µg/kg reversed AP200 to control level of 95 ± 1% (n = 10 rats). Similar effect was observed after IP injection of pyridostigmine, 100 µg/kg: reversal of AP200 to 95 ± 1% of AP1 in control (n = 10 rats).

**Figure 3:**
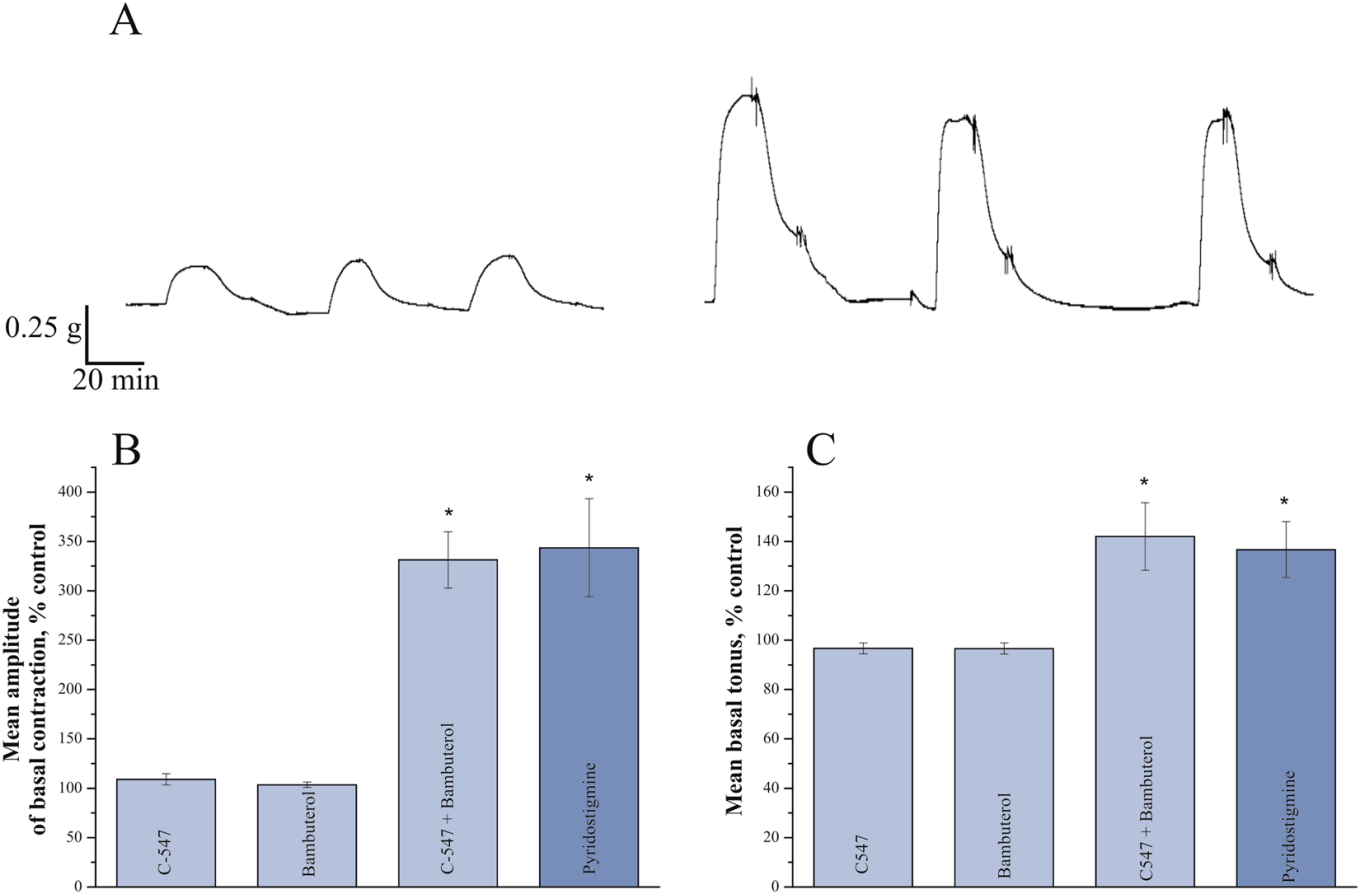
Co-inhibition of acetylcholinesterase and butyrylcholinesterase increases urinary bladder contractions recorded *in vivo* in rats affected by EAMG. (**A**) Representative individual urinary bladder basal contractions recorded in the same rat with intact cholinesterases (left) and after IP injection of non-specific cholinesterase inhibitor pyridostigmine at the dose of 0.1 mg/kg (right). (**B**) Mean amplitude of urinary bladder basal contractions recorded in diseased animals following inhibition of cholinesterase activity: acetylcholinesterase - by C547 (0.008 mg/kg), butyrylcholinesterase - by bambuterol (0.2 mg/kg) and both cholinesterases - by C547 together with bambuterol or by non-specific inhibitor pyridostigmine (0.1 mg/kg). (**C**) Mean basal tonus recorded in the same animals as amplitude of urinary bladder basal contraction. Data are presented as mean ± SEM pooled from ten animals in each group. Asterisk (*) indicates statistically significant difference (p < 0.05).

Then we measured tonus and amplitude of spontaneous contractions in urinary bladders in diseased rats. Pyridostigmine at 100 µg/kg, IP significantly increased basal tonus of the bladder (to 137 ± 11%; n = 10 rats, p = 0.02, Mann-Whitney test). In addition, administration of pyridostigmine led to significant increase in the amplitude of spontaneous contractions (to 345 ± 50%; n = 10 rats, p = 0.01, Mann-Whitney test). In contrast to pyridostigmine, C547, 8 µg/kg, IP had no significant effect on either basal tonus or amplitude of spontaneous contractions (n = 10 rats; Fig. 3).

It was previously shown that both AChE and BChE are involved in cholinergic modulation of rat urinary bladder contractions^18^ and guinea pig detrusor muscle tonus^19^. Thus, it could be hypothesized that the differences seen in this study are accounted for by the extent of inhibition of BChE by AChE-selective agent C547 (weak effect on BChE) and non-specific ChEs inhibitor pyridostigmine (strong effect on BChE). To test this assumption, highly selective BChE inhibitor bambuterol, 200 µg/kg, IP was administrated alone or after C547.

**Figure 4:**
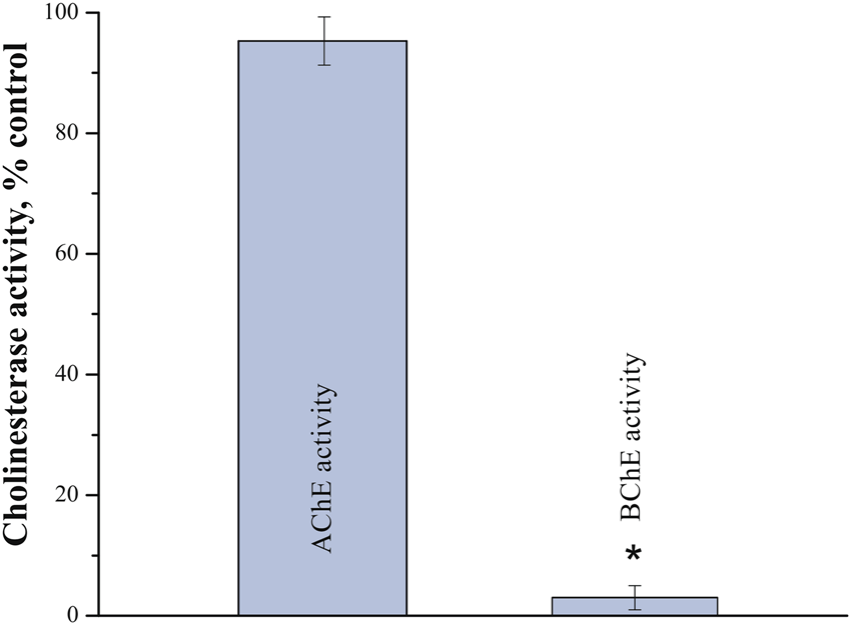
Bambuterol specifically blocks BChE in urinary bladder. Residual activities of acetylcholinesterase (AChE) and butyrylcholinesterase (BChE) were estimated in homogenates of rat urinary bladders after single IP injection of bambuterol at the dose of 0.2 mg/kg (six animals). Data are presented as mean ± SEM. Asterisk (*) indicates statistically significant difference (p < 0.05). AChE or BChE activity in homogenates of urinary bladders from control group (six animals) was taken as 100%.

As a first step, we decided to check-up how bambuterol affects the activity of BChE and AChE in homogenates of rat urinary bladder muscle. 20 min after IP administration of bambuterol no significant change in AChE activity was detected, while activity of BChE was inhibited by 98 ± 2% as compared to untreated animals (n = 6 rats *per* group; p = 0.001, Mann-Whitney test; Fig. 4).

Next, we found that bambuterol by itself had no significant effect on basal tonus and amplitude of spontaneous urinary bladder contractions (n = 10 rats). However, administration of bambuterol, after pre-treatment with C547, affected basal tonus and amplitude of contractions similarly to pyridostigmine, i.e., increasing the amplitude to 142.0 ± 13.7% *vs*. 136.7 ± 11.3% and to 331.3 ± 28.5% *vs*. 343.6 ± 49.6%, respectively (n = 10 rats; p < 0.02, Mann-Whitney test; Fig. 3).

Thus, higher selectivity of C547 to AChE *vs*. BChE, could account for its weaker effects in smooth muscle *in vivo* as compared to pyridostigmine. Tentatively one can assume that *in vivo*, BChE is able to significantly surrogate AChE function in urinary bladder, when AChE is partially inhibited by C547.

### Effect of C547 and pyridostigmine on the amplitude of contractions in rat and human urinary bladder strips *ex vivo*

Assuming, that unsuppressed BChE activity in urinary bladder can play a critical role in reducing side effects during pharmacological *MG* treatment, we decided to compare effects of AChE and BChE inhibition in rat and human urinary bladder preparations *ex vivo*. For that purpose, we have measured the amplitude of muscle contractions in bladder strips caused by application of ACh, 100 μM.

**Figure 5:**
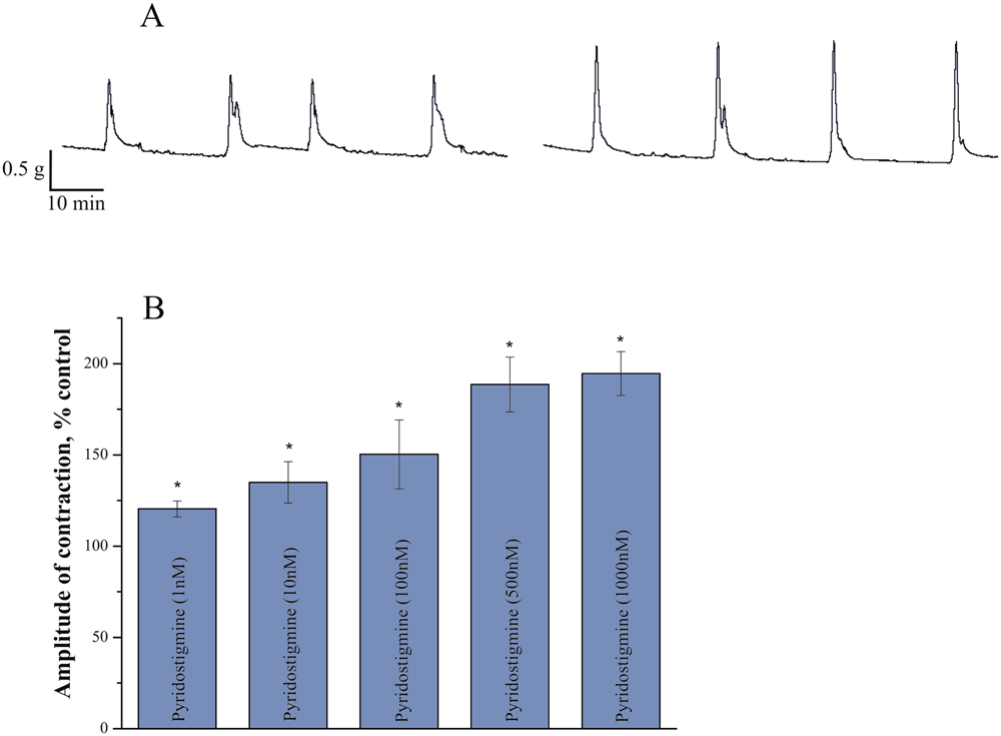
Augmentation of contractions in rat urinary bladder strips by pyridostigmine. (**A**) Typical contractions of rat urinary bladder strip induced by acetylcholine (100 μM) in control (left) and after inhibition of cholinesterases by 1 μM of pyridostigmine (right). (**B**) Dose-dependent effect of different pyridostigmine concentrations (1–1000 nM) on amplitude of ACh-induced contractions. Data are presented as mean ± SEM pooled from ten urinary bladder strips in each group. Asterisk (*) indicates statistically significant difference (p < 0.05).

We found that both pyridostigmine and C547 were able to increase the amplitude of ACh-induced contractions of rat urinary bladder strips in a dose-dependent manner. Each concentration for each drug was tested in 10 animals. Maximum effect of pyridostigmine was achieved at 1 µM (increase to 195 ± 8%; n = 10 preparations, p = 0.02, Mann-Whitney test; Fig. 5).

**Figure 6:**
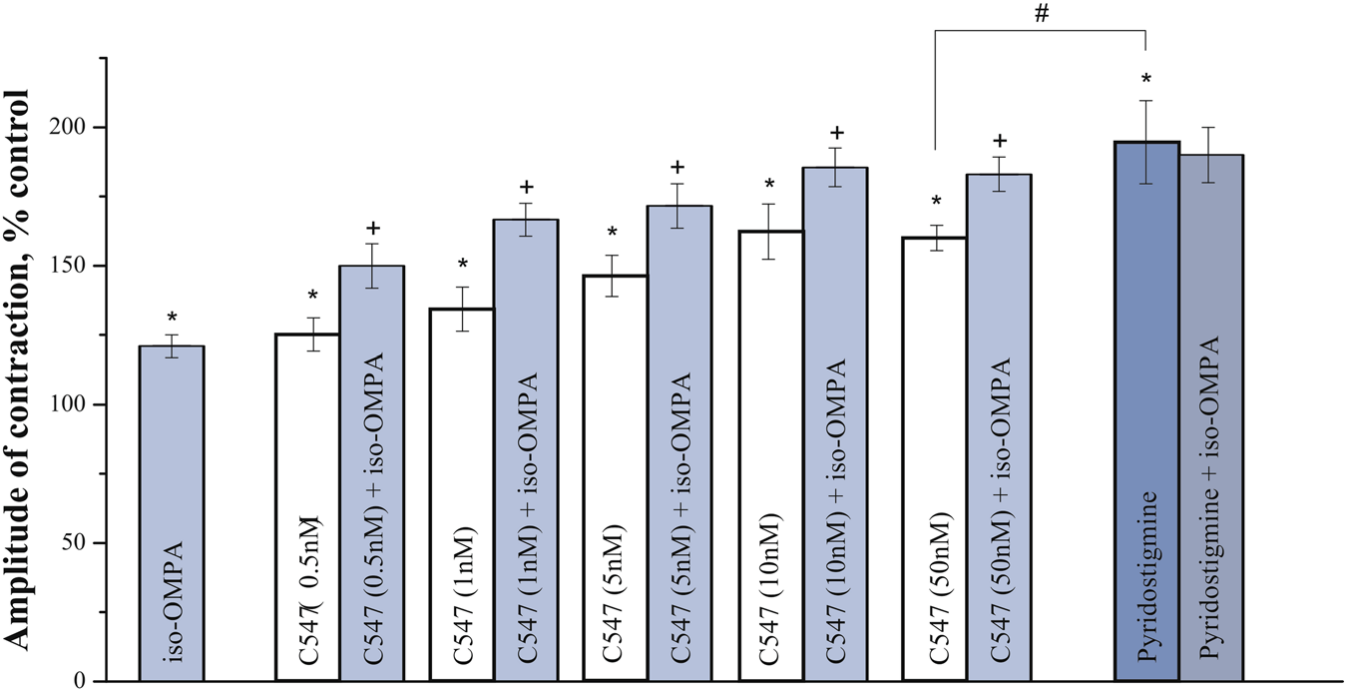
Additive effects of specific inhibition of BChE and AChE on amplitude of contractions of rat urinary bladder strips. Specific inhibition of BChE by iso-OMPA (50 μM) enhances ACh-induced contractions. Similar levels of potentiation of contractions (≈20%) were observed when iso-OMPA was applied alone or upon application of various concentrations of C547 (0.5–50 nM) – a specific AChE inhibitor. Iso-OMPA has no significant additional effect when applied after pyridostigmine (1 μM) – a non-specific cholinesterase inhibitor. Data are presented as mean ± SEM pooled from ten urinary bladder strips in each group. Asterisk (*) indicates statistically significant difference (p < 0.05) from control. Cross sign (+) indicates statistically significant difference (p < 0.05) in effects of combined application of iso-OMPA (50 μM) and C547 (0.5–50 nM) and application of any given single concentration of C457. Hash/pound sign (#) indicates statistically significant difference (p < 0.05) in effects of C547 (50 nM) and pyridostigmine (1 μM).

C547 also caused a significant increase in contractions starting at 0.5 nM (to 125 ± 6%; n = 10 preparations, p = 0.03, Mann-Whitney test) and reaching maximum effect at 10 nM (to 162 ± 10%; n = 10 preparations, p = 0.02, Mann-Whitney test). Maximum effect of C547 on muscle contraction was significantly lower than maximum effect of pyridostigmine. From our previous biochemical experiments we learnt, that at 10 nM C547 significantly inhibits AChE^13^, but not BChE; 1 μM of pyridostigmine completely blocks both ChEs^20^. Thus, one could hypothesize that it was just inhibition of BChE that accounts for the difference seen in the maximum effects of C547 and pyridostigmine. To prove this suggestion, we used iso-OMPA, a selective irreversible inhibitor of BChE, which completely inhibits BChE at 50 μM. We found that BChE inhibition alone or in combination with C547 increased the amplitude of ACh-induced contractions (Fig. 6). In a separate set of experiments, we have shown, that all the effects of ChEs inhibitors on muscle contraction in rat bladder strips were intermediated by activation of muscarinic acetylcholine receptors mAChRs, since these effects were completely eliminated by pre-incubation with muscarinic antagonist atropine, 1 μM (data not shown).

**Figure 7:**
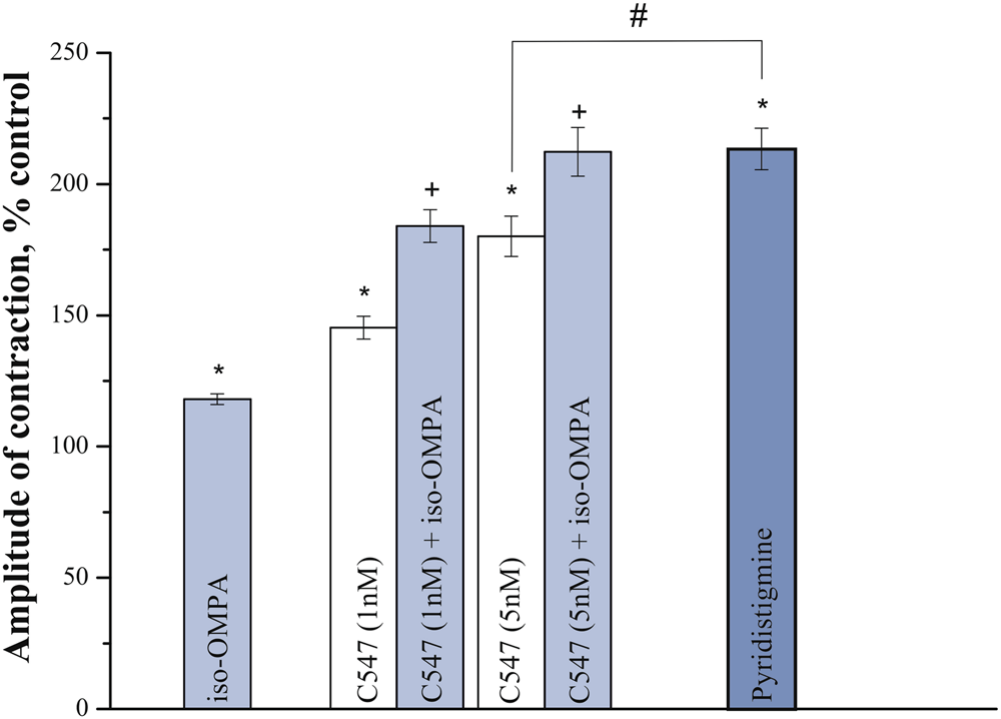
Effects of inhibitors of cholinesterase activity on contractility of human urinary bladder strips. The bar graph represents the effects of iso-OMPA (50 μM), C547 (1 and 5 nM) and pyridostigmine (1 μM) on contractions produced by acetylcholine (100 μM) in human urinary bladder strips. Data are presented as mean ± SEM pooled from four bladder strips in each group, obtained from ten patients. Asterisk (*) indicates a statistically significant difference (p < 0.05) from control. Cross sign (+) indicates statistically significant difference (p < 0.05) in effects of combined application of iso-OMPA (50 μM) and C547 (1 and 5 nM) and application of any given single C547 concentration of C547. Hash/pound sign (#) indicates statistically significant difference (p < 0.05) in effects of C547 (5 nM) and pyridostigmine (1 μM).

Next, we have studied effects of C547 and pyridostigmine on the amplitude of ACh-induced contractions in human urinary bladder strips. We used pyridostigmine in concentration of 1 μM which was expected to completely block both AChE and BChE and cause maximum effect on ACh-induced contractions. We found, that treatment with pyridostigmine markedly augmented the amplitude of ACh-induced contractions (Fig. 7). C547 was also found to increase contractions of human bladder strips in a dose-dependent manner. Concentration of 0.1 nM was ineffective (data not shown), whereas 1 nM C547 increased the amplitude significantly (to 145 ± 7%; n = 6 preparations, p = 0.03, Mann-Whitney test). At 5 nM C547 caused maximum effect (increase to 180 ± 7%; n = 6 preparations, p = 0.02, Mann-Whitney test), which was significantly lower than that of pyridostigmine (to 213.4 ± 8%; n = 6 preparations, p = 0.01, Mann-Whitney test).

In the next series of experiments, we studied the role of BChE inhibition in potentiation of contractions in human bladder strips. Selective, irreversible BChE inhibitor iso-OMPA (50 μM) was applied alone or after C547. Iso-OMPA by itself significantly increased bladder strips contractions (to 118.0 ± 2.0%; n = 6 strips, p = 0.02, Mann-Whitney test). Application of iso-OMPA after pre-treatment with C547 caused a further increase in contraction in an additive manner, and combined maximum effect reached the level of maximum effect caused by pyridostigmine (Fig. 7). It is worth noting that sensitivity of human bladder strips to BChE inhibition was found to be similar, if not identical, to sensitivity of rat bladder preparation. This is a very encouraging result and it would suggest that in human bladder, when treated with selective AChE inhibitors, the residual activity of BChE could be also sufficient to guarantee a significant reduction in side effects related to hyperactivation of smooth muscles.

## Discussion

Mammalian family of ChEs includes AChE (E.C. 3.1.1.7) and BChE (E.C. 3.1.1.8) which arе the enzymes hydrolyzing ACh and thus controlling its lifetime and interaction with postsynaptic nAChRs. For symptomatic treatment of muscle weakness, partial inhibition of ChE activity is used in clinic, when the disease is accompanied by reduction in the density of functional nAChRs in NMJs.

One of the main objectives of this study was to compare effects of C547, a novel, selective inhibitor of AChE, and pyridostigmine, a traditional, clinically used non-selective inhibitor of ChEs, on hyperactivation of smooth musculature in urine bladder of EAMG rats. Hyperactivation represents one of the major side effects when ChEs inhibitors are used for treatment of *MG* in human patients. To accomplish this, we used the method developed to measure urinary bladder contractile force *in vivo* ^21^.

In the EAMG model used, administration of pyridostigmine at a dose alleviating *MG* symptoms caused significant increase in the force of spontaneous contractions and enhancement of bladder tonus. At the same time, equi-potent dose of C547 did not affect the force of spontaneous contractions and basal tonus of rat urinary bladder. Since the most obvious pharmacological difference between pyridostigmine and C547 is the higher AChE selectivity of the latter, we suggested that background activity of BChE in the bladder could be sufficient to compensate for partial inhibition of AChE, in contrast to skeletal muscle. To test this hypothesis we administered selective BChE inhibitor, bambuterol, at a dose causing 98% block of BChE. In these conditions, almost complete inhibition of BChE did not affect either the force of contractions or tonus of EAMG rat bladders *in vivo*. These data suggest that under normal conditions BChE probably does not contribute significantly to ACh hydrolysis in rat urinary bladder. In contrast, one can assume that AChE is the main enzyme, controlling lifetime of ACh in urinary bladder of normal rats.

Following C547 pre-administration, bambuterol significantly increased the force of contractions and tonus of rat bladder to the extent similar to pyridostigmine. This fact implies that, in spite of its secondary role, the activity of BChE in the bladder could be sufficient to compensate for partial AChE inhibition, when C547 is used at the doses effective in alleviation of *MG* symptoms.

Functional role of BChE could be different in the urinary bladder of rat and human. To check-up whether activity of BChE in human urinary bladder will be also sufficient to reduce/diminish side effects of selective AChE inhibitors, we compared ACh-induced muscle contractions in rat and human bladder preparations *ex vivo* in the presence of AChE and BChE inhibitors. We found that sensitivity of these two preparations to inhibition of AChE and BChE was very similar. Thus, one can assume that preservation of certain level of BChE activity in human bladder can also help to decrease hyperactivation of smooth muscle and reduce related side effects during *MG* therapy. On the scientific venue, the results of this study warrant further investigation of the effects of inhibitors of AChE and BChE in human smooth muscles. For example, it would be interesting to compare activities of BChE at therapeutically effective doses of C547 and pyridostigmine in human bladder preparations.

We would like to emphasize some other important aspects of using AChE-selective inhibitors to treat *MG*. Pharmacokinetics of specific AChE inhibitors should be obviously distinct from that of non-specific inhibitors because BChE is highly abundant in serum and other tissues^22^. Indeed, BChE is a scavenger of non-specific ChE inhibitors and should decrease their active concentration around AChE. The levels of BChE vary in human populations due to genetic polymorphism and that is why the use of non-specific inhibitors is more difficult since they bind to variable levels of BChE as compared to specific inhibitors of AChE.

It has been shown earlier that at the mouse NMJs, BChE inhibition does not enhance activation of muscle type nAChRs by synaptically released ACh^23^. Also, it has been found that BChE, together with neuronal type α7 nAChRs, is localized at the terminal Schwann cells. These receptors sense spillover of ACh and their interaction with spilled ACh is predominantly controlled by BChE. Inhibition of BChE enhances activation of α7 nAChR and significantly depresses synaptic release of ACh^15^. Thus, when non-selective ChE inhibitors are used for *MG* treatment, blockage of BChE can counteract positive influence of AChE inhibition at the synapses of striated muscles.

The repertoire of pharmacological inhibitors of ChEs for treatment of muscle weakness in *MG* patients remains poorly developed. Currently, the two most frequently used inhibitors are pyridostigmine and neostigmine. They both suffer from side effects stemmed from hyperactivation of smooth muscles in urinary bladder and intestine. Pyridostigmine is a pseudo-reversible carbamylating agent, its selectivity for human AChE *vs*. BChE is very poor^20^. Neostigmine (dimethyl carbamate, analog of pyridostigmine) is also pseudo-reversible non-specific ChEs inhibitor. Neostigmine is used less frequently since it has stronger muscarinic side effects than pyridostigmine^24^. Thus, there is a clinical need in a new generation of ChEs inhibitors and an expansion of the assortment of selective AChE *vs*. BChE inhibitors designed for *MG* treatment is of undisputed interest.

We can conclude, that selective inhibitors of AChE may have a clinical advantage over non-selective ChEs inhibitors when used for *MG* treatment, particularly due to safer side effect profile related to urinary bladder hyperactivity.

## Materials and Methods

### Chemicals

1,3-bis[5(diethyl-*o*-nitrobenzylammonium)pentyl]-6-methyluracil dibromide (C547) was synthesized in the A. E. Arbuzov Institute of Organic and Physical Chemistry, Kazan, Russia.

Bambuterol, pyridostigmine bromide, tetra-isopropyl pyrophosphoramide, [1,5-bis (4-allyldimethylammo-niumphenyl) pentan-3-one dibromide], acetylthiocholine chloride, acetylcholine chloride, (5,5′-dithiobis-(2-nitrobenzoic acid) were purchased from Sigma-Aldrich.

Peptide DGDFAIVKFTKVLLDYTGHI was synthesis by “ATG Service Gene” company (St. Petersburg, Russia), quality of peptide synthesis was controlled by HPLC.

### Endplate electrophysiology

Experiments involving animals were performed in accordance with the guidelines set forth by the European Communities Council Directive of November 24, 1986 (86/609/EEC) and the experimental protocol approved by the Animal Care and Use Committee of the Kazan State Medical University.

Rat EDL muscle were fixed in translucent chambers and superfused with oxygenated Ringer-Krebs rat solution with the following composition (mM): NaCl 120.0, KCl 5.0, CaCl_2_ 2.0, MgCl_2_ 1.0, NaHCO_3_ 11.0, NaH_2_PO_4_ 1.0, glucose 11.0 рН 7.2–7.4. Resting membrane potential and miniature endplate currents (mEPCs) were recorded in the synaptic zone at 20–22 °C, using standard microelectrodes (resistance range of 10–15 MΩ when filled with 3 M KCl), Axoclamp 900 A amplifier (Molecular Devices, USA) and Digidata 1440 A (Axon Instruments, USA) with WinWCP software (John Dempster, University of Strathclyde, United Kingdom). In the voltage-clamp mode membrane potential was held at −60 mV.

C547 or pyridostigmine were applied in Ringer-Krebs solution.

Data were expressed as the mean ± SEM. Statistical analysis was performed using the unpaired Student’s t-test. P < 0.05 was considered statistically significant.

### *MG* animal model and *in vivo* urinary bladder contraction studies

Experimental autoimmune *MG* was induced in rats using the following protocol^14^. Female Wistar rats 6–8 weeks of age were immunized twice (1 month interval) by subcutaneous administration of the peptide (DGDFAIVKFTKVLLDYTGHI) mixed in Freund’s complete adjuvant (first injection) and incomplete adjuvant (second injection). Development of muscle weakness was diagnosed *in vivo* by the marked decrement in the amplitude of surface electromyogram (compound AP) of hindlimb muscles^15^. Under urethane anesthesia (1.2 g/kg, IP), the sciatic nerve was stimulated (40 Hz, train of 200 stimuli) in the femoral part. The compound muscle AP from posterior surface of the lower leg was recorded *via* skin electrodes. Compound muscle AP was recorded using FE132 amplifier (ADInstruments, Australia) and digitized with the help of PowerLab4/35 system (ADInstruments).

Polyethylene catheter (PE 50; Instech Laboratories, USA) was inserted into the bladder of *MG* rats through urethra and secured by a ligature. The urinary bladder was filled with physiological saline (0.2–0.7 mL) and connected to a pressure transducer (MLT844, ADInstruments) for intraluminal pressure recording with the LabChart data acquisition system (ADInstruments). Animals were allowed to equilibrate under anesthesia, before baseline level and amplitude of spontaneous contractions stabilized. Then an aliquot of physiological saline, as control, or drug-containing solutions were intraperitoneally administered and bladder response was recorded. Basal tonus and amplitude of spontaneous contraction were analyzed.

C547, bambuterol and pyridostigmine dissolved in physiological saline were delivered intraperitoneally (IP).

Data were expressed as the mean ± SEM. Urinary bladder contractions after drug administration were expressed as percentage of contractions in control. Statistical significance was proven by Mann-Whitney test at the level of P < 0.05.

### Cholinesterase inhibition assay

For these studies, rat urinary bladders were surgically excised 20 min after single IP injection of 200 µg/kg bambuterol (experimental group, 6 rats) or after 0.9% NaCl IP injection (control group, 6 rats). Tissues were frozen in liquid nitrogen. Homogenates of whole urinary bladder were prepared at a ratio of 1:4 in a Potter homogenizer with 0.05 M Tris-HCl, 1% Tween-20, 1 M NaCl, 2 mM EDTA; pH 7.0, at 4 °C. Homogenates were centrifuged (at 10 000 rev/min, 4 °C) for 10 minutes using Eppendorf 5430 R centrifuge with FA-45-30-11 rotor (Eppendorf AG, Hamburg, Germany).

ChEs activity was measured by the method of Ellman^25^ based on production of yellow 5-thio-2-nitro-benzoate anion detected by PerkinElmer λ25 spectrophotometer at 412 nm in modification of Dingova *et al*.^26^. For AChE activity assay, 50 µl of supernatant were incubated with 5 µl of BChE inhibitor, tetra-isopropyl pyrophosphoramide (iso-OMPA), at the final concentration of 50 µM, for 30 minutes. For BChE activity assay, 50 µl of supernatant were incubated with 5 µl of AChE inhibitor, [1,5-bis (4-allyldimethylammoniumphenyl) pentan-3-one dibromide] (BW284c51), at the final concentration of 1 µM, for 30 minutes. After that, the enzyme-catalyzed hydrolysis reaction was started by adding 10 µl of acetylthiocholine (final concentration of 1 mМ) as substrate. After 10, 20 or 30 min incubation at 25 °C reaction was stopped by adding neostigmine (0.1 mM). Samples were diluted in 50 mM phosphate buffer (pH 8.0) and DTNB (0.1 mM) was added. The rate of thiocholine production during 20 min (10th–30th min) was calculated. Cholinesterase activity in bladder samples from control group was assumed to be 100%. Sample without substrate was used as a blank. All measurements for each sample were triplicated. AChE or BChE activity was expressed in relation to the amount of total protein, which was determined by the Bradford method^27^. Data were analyzed using Origin 8 and expressed as the mean ± SEM. Statistical analysis was assessed by Mann-Whitney test. P < 0.05 was considered statistically significant.

### Urinary bladder strips contraction

The longitudinal strips of rat or human urinary bladder were mounted in 5 mL double jacketed organ baths containing oxygenated Krebs (mM): NaCl 120.0, KCl 5.0, CaCl_2_ 2.0, MgCl_2_ 1.0, NaHCO_3_ 11.0, NaH_2_PO_4_ 1.0, glucose 11.0, рН 7.2–7.4 at 25 °C. The strips were allowed to equilibrate for at least 1 h, during which resting tension of 0.5 g was applied. Isometric tension changes were measured using TRI201 tension transducers and PowerLab 4/35 system (ADInstruments). All preparations were contracted with ACh, 100 μM. Then the preparations were washed with fresh Krebs solution and allowed to return to their resting tone. Contractions were induced every 30 min. When amplitude of contractions was stabilized, experimental drugs were added to the bath. Preparations were incubated for 30 min in Krebs solution containing different concentration of ChEs inhibitors, and subsequently ACh-evoked contraction was recorded.

Human bladder muscles were obtained in Clinic of the Kazan Medical University from ten Caucasian patients undergoing transvesical prostatectomy for benign prostatic enlargement, after receiving informed consent. The study was approved by the ethical committee of the Kazan Medical University according to Russian national ethical guidelines. No organs/tissues were procured from prisoners. Patient anonymity was strictly maintained. In these patients, a strip of muscle was taken from the anterior wall of the dome of the bladder by making the incision into the bladder. None of the patients undergoing operative surgery received anticholinergic drugs (e.g. atropine) or adrenergic blockers, either in pre-medication or during surgery.

Data were expressed as the mean ± SEM. Drug effect was expressed as percentage of ACh-induced contraction amplitude in control. Statistical significance was assessed by Mann-Whitney test at the level of P < 0.05.
